# Effect of Natural Graphite Fineness on the Performance and Electrical Conductivity of Cement Paste Mixes for Self-Sensing Structures

**DOI:** 10.3390/ma13245833

**Published:** 2020-12-21

**Authors:** Ioanna Papanikolaou, Chrysoula Litina, Amir Zomorodian, Abir Al-Tabbaa

**Affiliations:** Department of Engineering, University of Cambridge, Trumpington St., Cambridge CB2 1PZ, UK; cl519@cam.ac.uk (C.L.); az412@cam.ac.uk (A.Z.); aa22@cam.ac.uk (A.A.-T.)

**Keywords:** cement, graphite, additives, electrical conductivity, self-sensing

## Abstract

Cementitious composites are the most widely used construction materials; however, their poor durability necessitates frequent monitoring and repairs. The emergence of self-sensing composites could reduce the need for costly and time-consuming structural inspections. Natural graphite, due to its low cost and wide availability, is a promising additive to generate an electrically conductive network which could ultimately lead to a self-sensing mechanism. Despite several studies using natural graphite as a conductive additive, the effect of its fineness on the cementitious composite’s performance has not been explored. This study experimentally investigated the effect of three graphite products of varying fineness on the early age, mechanical, and electrical conductivity performance of cement pastes. The fluidity of the graphite-cement paste reduced significantly with increasing graphite fineness, and graphite did not affect the cement hydration. The finer the graphite, the lower the effect on the mechanical performance, as confirmed by compressive strength testing and micro-indentation. Electrical conductivity testing showed that the percolation threshold depended on the graphite fineness and was found at ~20 wt % for the fine and medium graphite, while it increased to 30–40 wt % for the coarse graphite. This is the first study that has investigated holistically the effect of graphite fineness on the performance of cement pastes and will pave the way for using this material as an additive for self-sensing structures.

## 1. Introduction

Cement composites, including pastes, mortars, and concrete are the most widely used construction materials; however, cementitious structures often suffer from poor durability that necessitates repair and maintenance (R&M) to be carried out. From 2011 to 2015, approximately a fifth of the civil engineering works in the UK were due to R&M [1], while in the US, Americans undertake over 200 million trips a day across deficient bridges [2]. This inadequate durability has resulted in a need for structural inspections which are usually carried out visually [3,4]; however, due to the inherent uncertainties and risks with visual surveys, the use of sensors has started to flourish. Despite the increasing use of structural health monitoring systems, the use of external sensors has often resulted in high costs, low sensitivity, need for frequent calibration, and incompatibility with structural materials [5,6,7].

Recent advances in material science have generated increasing interest in the use of smart, biomimetic materials that mimic natural systems and could monitor their own condition [4]. One such example, is self-sensing concrete, which refers to a material that can sense its condition and identify any damage, whilst maintaining or improving the structural performance [8]. A self-sensing mechanism can be achieved by passing electric current through the structure and monitoring changes in the electrical conductivity. The non-reversible change in conductivity can be used to monitor damage, while the reversible change can be used to monitor strain (loading) [8,9,10]. Nevertheless, cementitious composites behave as insulators to electricity and do not allow the passage of electric current [11]. Thus, the use of a conductive additive is necessary to ensure that an electrically conductive network can be formed within the cementitious structure.

In terms of conductive additives, at least 10 different types, along with hybrid combinations, have been investigated, such as steel fibers, carbon fibers, graphite powder, carbon nanotubes, and graphene nanoplatelets [8,12,13]. The minimum additive dosage that is needed to form continuous electrical paths inside the composite is known as the percolation threshold, which depends on many parameters, including additive composition (size and shape), concentration, and degree of aggregation [12]. For example, fibrous additives with a high aspect ratio, reach a percolation threshold at a lower dosage (~1.5 wt %), compared to particle ones (>5 wt %) [8].

One such functional additive is natural graphite, which due to its wide availability and low cost, could have promising applications in cementitious composites for self-sensing structures. Graphite powder has a layered planar structure, rendering it relatively soft due to its anisotropy and weak inter-planar forces, ability to conduct electricity and heat well, resistance to chemical attack, and stability under standard conditions [8,14]. Graphite has been used as a conductive filler in some studies, and it was found that it could improve the electrical conductivity performance [15,16,17,18]. However, graphite size can vary, and very often, this property is not reported, while at the same time, the percolation threshold could depend on the size of the conductive additive. In one study, it was found that when graphite was added in cement, the composite became a conductor; however, the mechanical and electrical properties depended on the water content and the setting process [15]. When dry-mixing graphite and cement powder, a minimum threshold of 2 wt % graphite was needed, below which the insulating cement prohibited the formation of conductive graphite pathways and the conductivity leveled off at around 10 wt % graphite [16]. Similarly, the DC electrical resistivity decreased rapidly between the percolation threshold at 2 and 10 wt %, where it plateaued with a graphite that had a particle size of 10–20 µm [17]; however, another study found a conductivity threshold at ~30 to 40 wt % of graphite with a *d*_50_ size of 4.5 µm [18].

Despite some promising early findings in the literature around the improvement in electrical conductivity with graphite, the effect of its fineness has not been investigated in detail. Most studies do not report the inherent material properties, such as the size; thus, it is not possible to understand the effect of the additive on the properties of cement composites and use the graphite material at its full potential. Furthermore, there is a lack of a holistic assessment of the effect of natural graphite, not only on the electrical conductivity, but also on other properties that are essential for cementitious composites, such as rheology, hydration, and mechanical performance. Therefore, this study experimentally investigated three natural graphite products of varying sizes to understand the effect of graphite fineness on the electrical conductivity, microstructure, hydration, and mechanical performance of cementitious pastes that could then be used for self-sensing applications.

## 2. Materials and Methods

### 2.1. Materials

Portland Cement CEMI 52.5N, supplied by Hanson and conforming to BS EN 197-1:2011 [19] was used and its chemical composition is tabulated in Table 1. The cement had a size distribution of 5–30 µm, a surface area of 0.3–0.4 m^2^/g, a density of 2.7–3.2 g/cm^3^ and a loss on ignition (LOI%) of 2.2, as provided by the supplier in the material safety data sheet. Three commercial products of natural graphite were used to create the graphite-cement paste. A coarse graphite powder (−10 mesh, 2 mm) was supplied by AlfaAesar (Haverhill, MA, USA), and two finer graphite powders (−100 mesh, 0.150 mm and −325 mesh, 44 µm) were supplied by Sigma-Aldrich (St. Louis, MO, USA). The suppliers only provided the mesh size of the graphite rather than the particle size distribution and hence only the mean size is used here. These graphite products are referred to as coarse (2 mm), medium (0.15 mm) and fine (44 µm). The graphite powders were used at varying concentrations needed to reach the percolation threshold (from 0 to 40 wt %). The cement paste had a constant *w/c* = 0.45. It should be noted that the graphite powders were added as additions rather than cement replacement; therefore, the cement content and *w/c* ratio are the same in all mixes.

### 2.2. Sample Preparation

To prepare the cement paste mixes, the graphite powder and cement were dry-mixed first, in a laboratory bench-scale mixer, for 2 min, and then water was added. Immediately, when the water was added, the mixer was started at a slow speed for 60 s and high speed for 30 s. Then, the mixer was stopped for 90 s, and during the first 30 s, the paste adhering to the walls and bottom of the bowl was removed with a scraper and placed in the middle of the bowl. Mixing was then continued at high speed, for a further 60 s. It should be noted that no chemical admixtures were used to aid the dispersion of the graphite. When mixing was completed, the fresh paste was cast in three layers of equal thicknesses, in oiled stainless-steel moulds. For compressive strength, the specimens had a size of 40 × 40 × 40 mm^3^; for micro-indentation, the cylindrical specimens were 50 mm diameter and 100 mm height; and for electrical conductivity, the prismatic specimens had a size of 40 × 40 × 160 mm^3^. The specimen size was reduced by half (20 × 20 × 80 mm^3^) for electrical impedance spectroscopy testing. The specimens were demolded after 24 h and cured in a water tank, at temperatures of 20 ± 2 °C and a relative humidity 60%, until testing.

### 2.3. Experimental Programme

#### 2.3.1. Material Characterization, Dispersion, and Microstructure

To assess the dispersion of the natural graphite in the cement paste and the microstructure of the composite, Scanning Electron Microscopy (SEM) was carried out, using a ZEISS EVO LS 15 SEM–EDX (Oberkochen, Germany). Chipped pieces were extracted from the cracked specimen surfaces, following the compressive strength testing, and all samples were gold-coated before testing.

Thermogravimetric analysis (TGA, PerkinElmer, Waltham, MA, USA) was used to characterize the graphite powder materials and understand their decomposition. The TGA experiment was performed in air. The temperature ranged from 40 to 1000 °C, at a steady rate increase of 10 °C/min, and the gas flow rate was kept constant at 30 mL/min.

X-ray computed tomography in the form of micro-CT scanning (μCT) was carried out, using a XT H 225 ST CT scan device by Nikon (Tokyo, Japan). A ~5 mm sample was extracted from the cracked surface of the cement paste specimens containing natural graphite and analyzed.

#### 2.3.2. Rheology Measurement

To assess the rheology of the graphite-cement pastes, a smooth-walled Brookfield DV3T Rheometer (SC4-27 spindle, Middleboro, MA, USA) was used. Measurements were carried out every 15 s at room temperature and a shearing profile that is representative of the typical concreting process, such as hauling and casting, was followed [20]. Each sample was pre-sheared for 1 min, and it was then left for 30 s, to stabilize. The shear speed increased progressively from 0 to 150 rpm, in 25 rpm intervals. It was then kept constant at 175 rpm and then decreased progressively from 150 to 0 rpm, again, in 25 rpm intervals (descending rates). The Bingham model was used to calculate the viscosity of the samples, using the descending shearing rates.

#### 2.3.3. Hydration, Compressive Strength and Micro-Indentation

An isothermal calorimeter I-CAL 2000 HPC was used and ASTM C1679-08 [21] was followed to assess the effect of natural graphite on the hydration of cement paste. A CONTROLS Advantest9 machine (Milan, Italy) was used for the unconfined compressive strength at a loading rate of 2400 N/s according to BS EN 196-1 [22], and three cubic specimens of 40 × 40 × 40 mm^3^ were tested at 2, 7, and 28 days. Micro-indentation testing was also performed to establish the effect of graphite on the hardness and elastic modulus of the specimens according to ASTM E384–16 [23]. Cylindrical specimens of 50 mm diameter and 100 mm height were prepared and cut into discs of ~25 mm thickness. The testing was carried out after 56 days of curing and only one concentration of each graphite was tested (20 wt %). To ensure a smooth surface, a polishing protocol was followed. A P240 silicon carbide (SiC) paper was first used for 30 s for both sides of the disc. One side was then polished progressively, using P400-P800-P2500 SiC paper, for 30 s in each stage. The polished discs were then tested with an Anton Paar MHT micro-indentation tester (Graz, Austria) with a Vickers tip. An indentation force of 8 N was used, and 15 measurements were taken for each sample following a protocol suitable for cement paste [24]. The loading and unloading times were 15 s, and the indentation time at maximum force (8 N) was 20 s, while a Poison’s ratio of 0.25 was used.

#### 2.3.4. Electrical Conductivity

**Figure 1:**
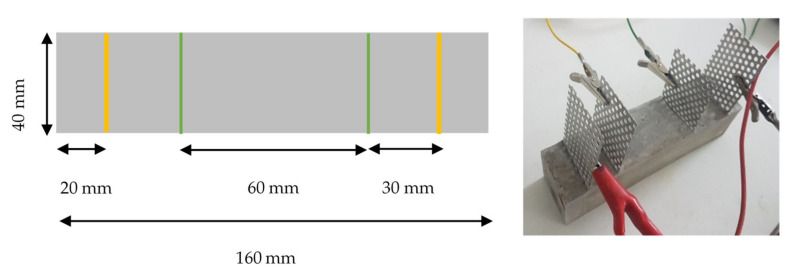
Four-probe setup for electrical resistivity measurements. Outer electrodes supplied a DC = 10 V, and inner electrodes measured the change in voltage.

The electrical conductivity was tested by using a 4-probe DC setup, using perforated steel sheet electrodes. A DC of 10 V was supplied to the outer two electrodes, and the inner two electrodes were used to measure the voltage (Figure 1), which was recorded with a datalogger every second and illustrated in the LABView software.

Electrical impedance spectroscopy (EIS) was used to characterize the electrical parameters of the materials and to investigate the frequency-dependent response and material–electrode interface. By applying an AC current with a set amplitude over a range of frequencies, the response of the specimen could be measured in terms of magnitude and phase angle. A 2-probe setup with embedded electrodes was used with specimens of 20 × 20 × 80 mm^3^ size. Two dosages were selected for each graphite type, one at 10 wt % and one at the start of the percolation threshold (either 20 or 40 wt % for the coarse graphite), and triplicate tests were run at 7 and 28 days. The results of the EIS testing are shown in the form of Nyquist plots, with the real part of the impedance shown in the *x*-axis (*Z*′(Ω)), and the imaginary part in the *y*-axis (−*Z*″(Ω)); the average of three samples is represented with each Nyquist plot. The Nyquist plot contains semicircles or arcs, and their diameters correspond to the resistances of the different components in the composite microstructure [25].

## 3. Results and Discussion

### 3.1. Material Characterization, Dispersion, and Microstructure

**Figure 2:**
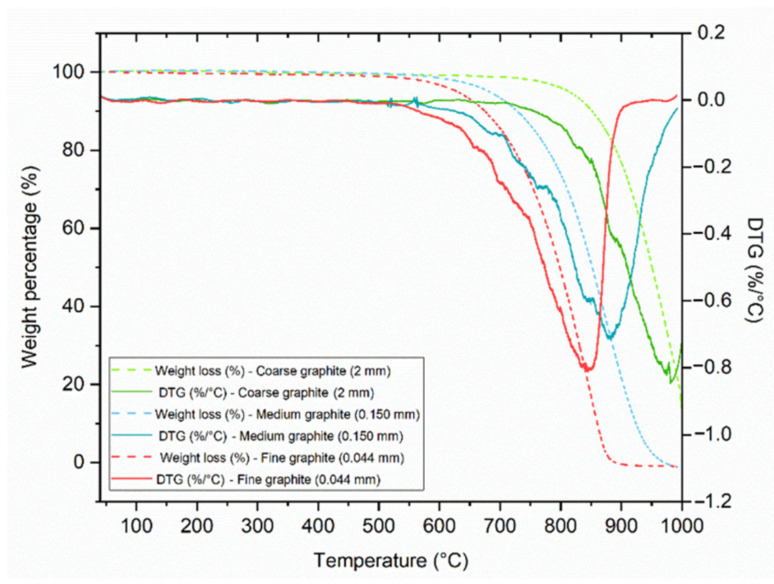
Characterization of the three graphite products by thermogravimetric analysis (TGA), showing the weight loss from 100 to 1000 °C.

The TGA results in Figure 2, show that the fine and medium graphite materials decomposed completely (lost 100% of their weight) between 40 and 1000 °C, while the coarse graphite did not. This could be due to the particle size of the coarse graphite (2 mm) as the flakes were too large for all their minerals to decompose. The smaller the particle size, the faster the minerals decompose with increasing temperature, and therefore the curves shift to a lower temperature as the material size reduces. None of the materials showed any mass loss before 100 °C; therefore, they are all expected to be stable during cement mixing and hydration. Furthermore, the DTG curves do not show the presence of any other minerals so the graphite products are of high purity.

**Figure 3:**
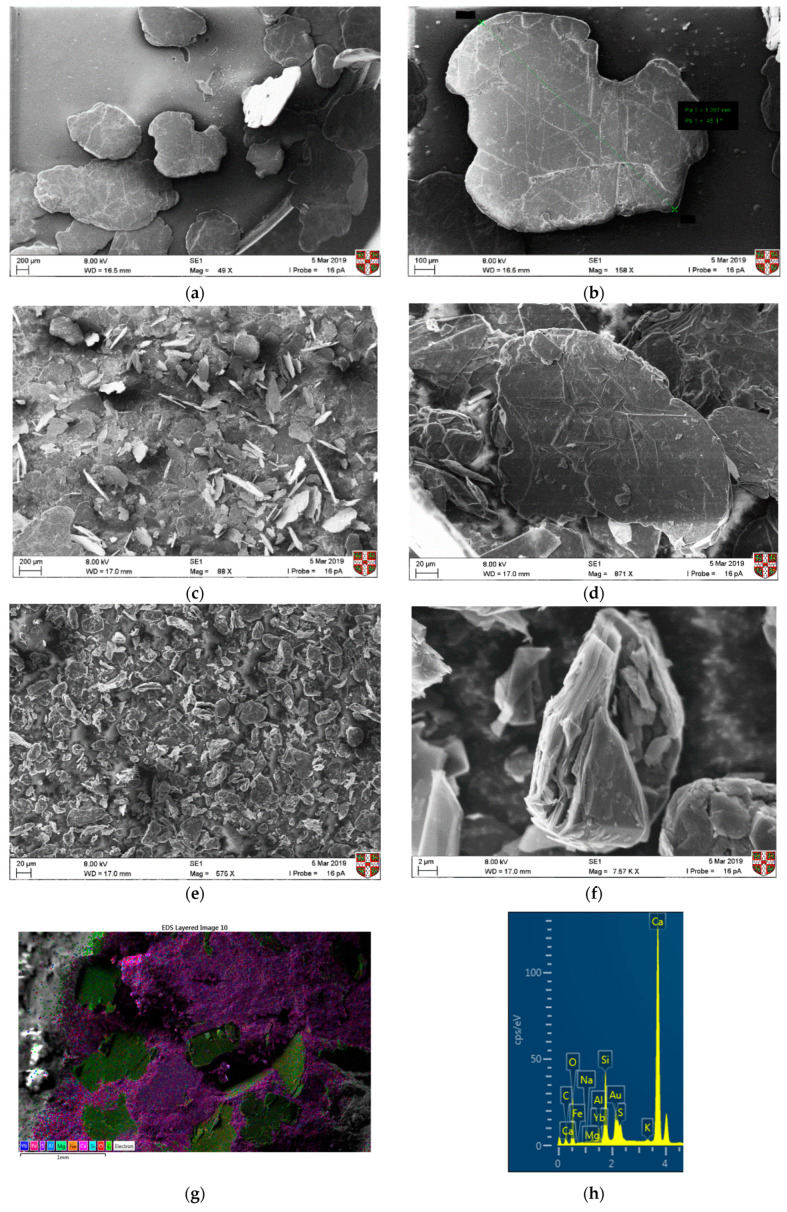
SEM images of the three graphite products, with two different magnification images for each: coarse graphite (**a**,**b**), medium graphite (**c**,**d**), fine graphite (**e**,**f**), and SEM–EDX confirming good dispersion of the coarse graphite flakes at 28 days (**g,h**).

SEM was used to understand the morphology of the graphite materials. In Figure 3a,b the coarse graphite and some large flakes with a size of at least 1 mm can be observed, which is expected due to the inaccuracies when sieving. For the medium graphite (Figure 3c,d), flakes are of varying sizes but are all between 150 and 200 µm. In Figure 3e,f, the fine graphite particles can be seen with a wrinkled and folded structure. As the graphite became finer, some agglomerates could be seen, and it was difficult to isolate individual flakes. SEM–EDX was also used to assess the dispersion effectiveness of the dry mixing protocol. Only a coarse graphite-cement paste specimen at 28 days of hydration was used and the coarse graphite particles are shown as green in Figure 3g, whilst the cement paste matrix is shown as pink. The EDS analysis (Figure 3h) confirms that the main elements are carbon and calcium, which was expected due to the presence of graphite and because calcium silicate (C–S–H) is the main reaction product of cement hydration. Other typical elements from cement hydration, such as silicon, oxygen, sulfur, and magnesium, were also present.

**Figure 4:**
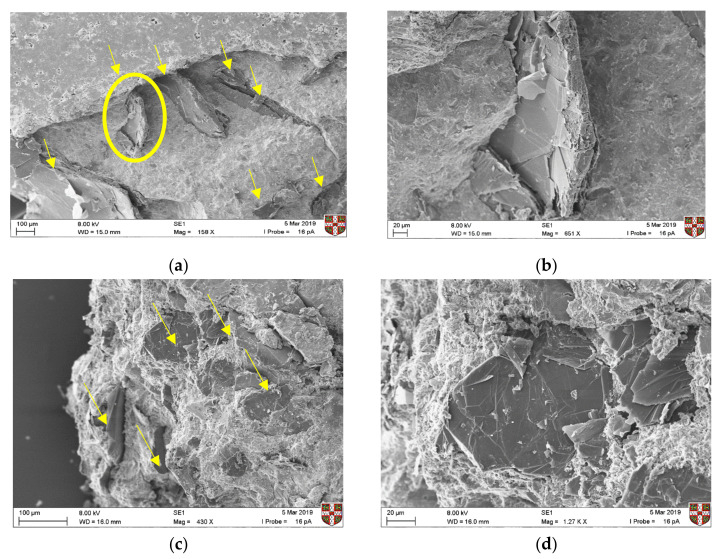

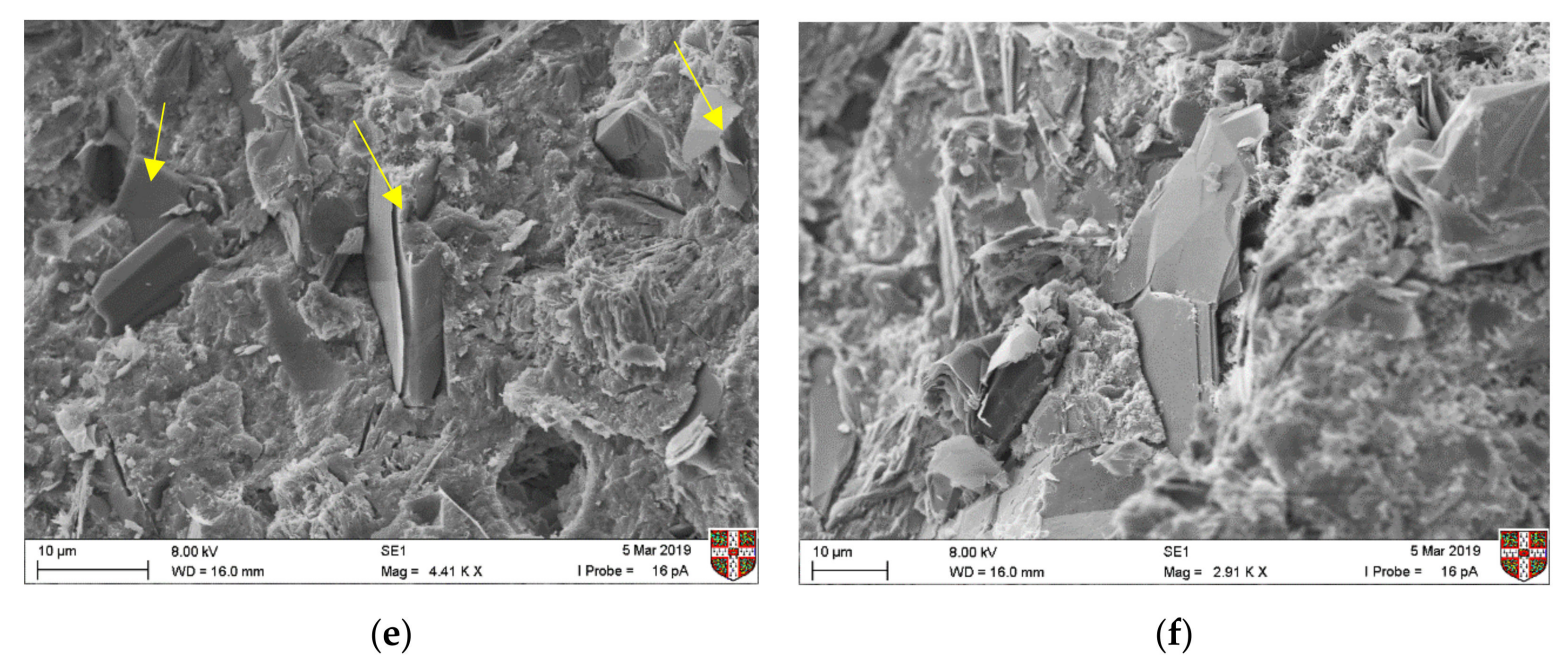
Microstructural characterization of the three graphites-cement paste after ~5 months by SEM (**a**,**b**) coarse, (**c**,**d**) medium, and (**e**,**f**) fine graphite.

SEM testing was also undertaken after five months of curing, to assess the interaction of the graphite particles with the hydrated cement paste and to understand the effect on microstructure. In Figure 4, the individual graphite flakes could be identified in all cases, irrespective of the graphite size. Flakes were observed in proximity, which was expected, as graphite concentrations were high (the percolation threshold samples are illustrated below), but no obvious agglomeration was present. Therefore, dispersion of graphite particles was assumed to be adequate with dry mixing, which is a technique that has also been followed in the literature [16]. Observing the interaction between the graphite particles and the cement hydration products, we can see an interfacial transition zone similar to that of aggregates for the coarse graphite (Figure 4a), which could lead to planes of weakness and a reduction in mechanical performance. These results are not directly comparable with other studies, as the microstructural interaction could also be affected by the cement type, dispersion technique and graphite morphology. Overall, none of the three natural graphite products that were used here was found to alter the microstructure of the CEMI paste.

**Figure 5:**
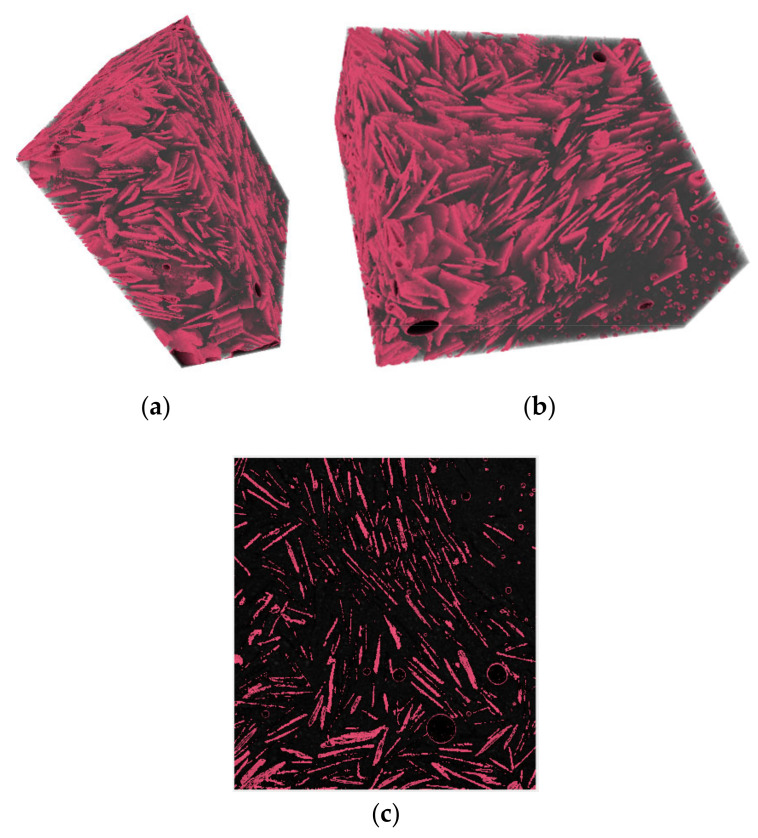
X-ray computed tomography (CT-scan) of an approximately 5 mm cement paste specimen with a 30 wt % coarse graphite addition. The graphite flakes (pink) are dispersed in the matrix (gray): (**a**,**b**) 3D reconstructed image of the specimen and (**c**) a slice through the 3D image.

A µCT-scan was also used to assess the dispersion of a 30 wt % coarse graphite dosage in the cement matrix with the dry mix protocol. Figure 5a,b shows the 3D reconstructed image of the 5 mm graphite-cement paste sample and Figure 5c shows a slice through the 3D image. A grayscale analysis was used to detect the different substances based on their density. Three distinct peaks were observed in the µCT-scan, with each representing the different elements including air, bulk cement paste, and graphite particles. In Figure 5, the graphite particles are represented with a pink color, whilst the bulk cement paste is shown as black. The graphite flakes were found to be well dispersed within the matrix and near each other which was due to the high graphite concentration (30 wt %). The orientation of the flakes appeared to vary, even though some clusters were oriented in the same direction. The key finding from the µCT-scan was that graphite flakes were well dispersed with the dry mix method, and even at a high graphite concentration, no agglomerates were observed.

The microstructural characterization showed that the dry-mixing protocol that was followed here was effective, while graphite had no pronounced effect on the microstructure of cement paste, irrespective of its fineness. This means that graphite is acting as an inert filler in the mix and does not alter the chemical structure of the hydrated cement paste.

### 3.2. Rheology of Graphite-Cement Pastes

**Figure 6:**
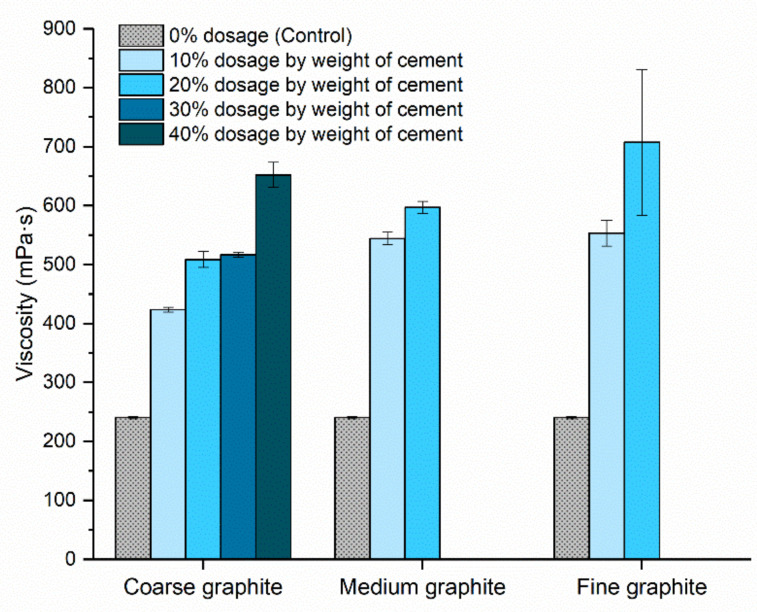
Effect of natural graphite size and concentration on the viscosity (mPa·s) of cement paste.

Rheology testing was carried out to assess the effect of graphite fineness on the fluidity of the pastes. A sufficient fluidity is necessary from a practical perspective for the material to be mixed, pumped, and placed without any bleeding or segregation. From Figure 6, the graphite addition resulted in an increase in the viscosity of cement paste in all cases. For the coarse graphite, the viscosity increased progressively by 76% to 171% compared to the control when graphite was added at concentrations of 10–40% by weight. For the medium graphite, viscosity also increased dramatically, and it was not possible to mix after the 20 wt % concentration. Specifically, the viscosity increased by 126% and 148% for the 10 and 20 wt % graphite, which was much higher than the coarse graphite at the same dosages. For the fine graphite at 10 wt %, the viscosity increase was comparable to that of the medium graphite but at 20 wt % dosage, the viscosity was almost three times higher compared to the control. Furthermore, the error bar was large at that concentration, due to difficulties in mixing this high fine graphite concentration. Due to the very high viscosity at the 20 wt % dosage, it was not possible to mix the higher 30 and 40 wt % concentrations with the fine graphite.

From rheology testing, it was found that increasing graphite fineness led to a dramatic reduction in fluidity, due to the inter-particle friction with cement particles, as well as because of the low hydrophilicity of graphite [26]. The finer the graphite, the more dramatic the reduction in fluidity (increase in viscosity) for a given % concentration; the fine graphite for the same weight dosage would have more particles that caused inter-particle friction with cement, and therefore the viscosity increased dramatically. Moreover, the smaller size graphite is expected to have a higher surface area, which would require more water to cover its surface. This effect on viscosity has also been confirmed in other studies that used additives in cementitious composites and can be explained by the *crowding* phenomenon, where an increase in the additive population obstructs the movement of water around them, thus increasing the viscosity [27,28]. Overall, the observed reduction in fluidity could introduce practical limitations when using graphite as a conductive additive, and the mix design and the water content would need to be adjusted, to ensure sufficient flowability.

### 3.3. Effect of Graphite on the Hydration of Cement Paste

**Figure 7:**
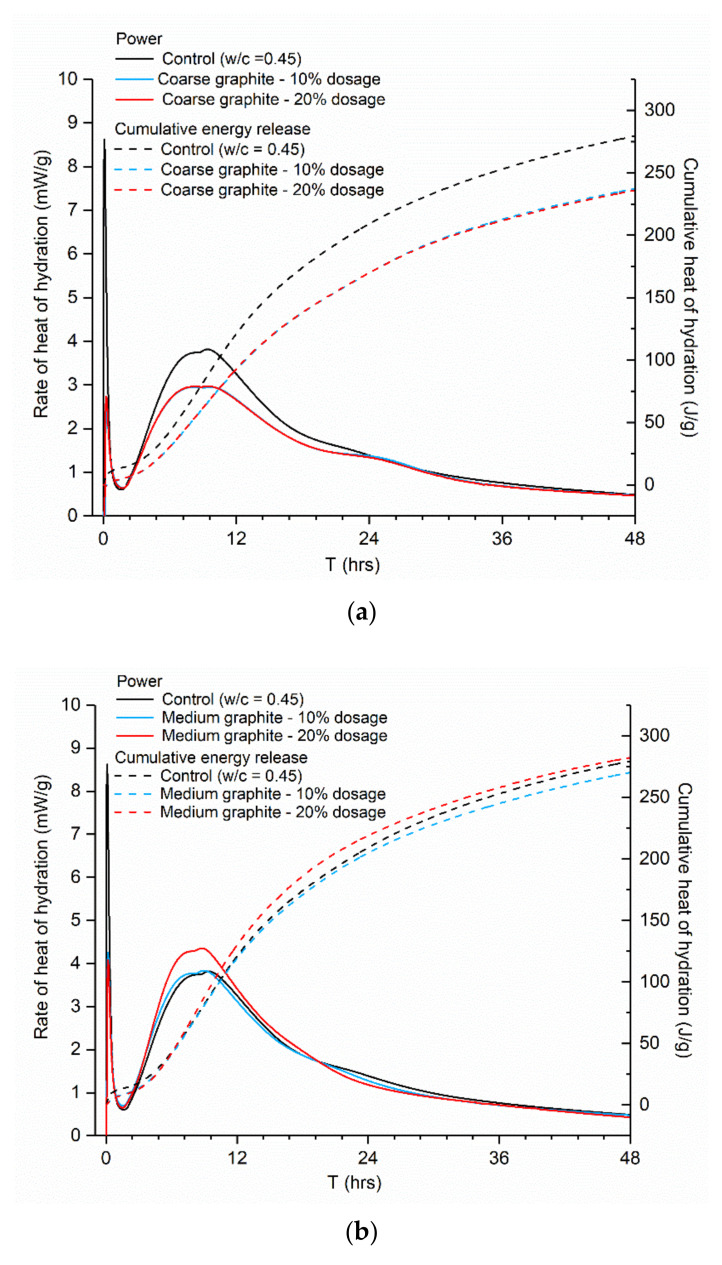

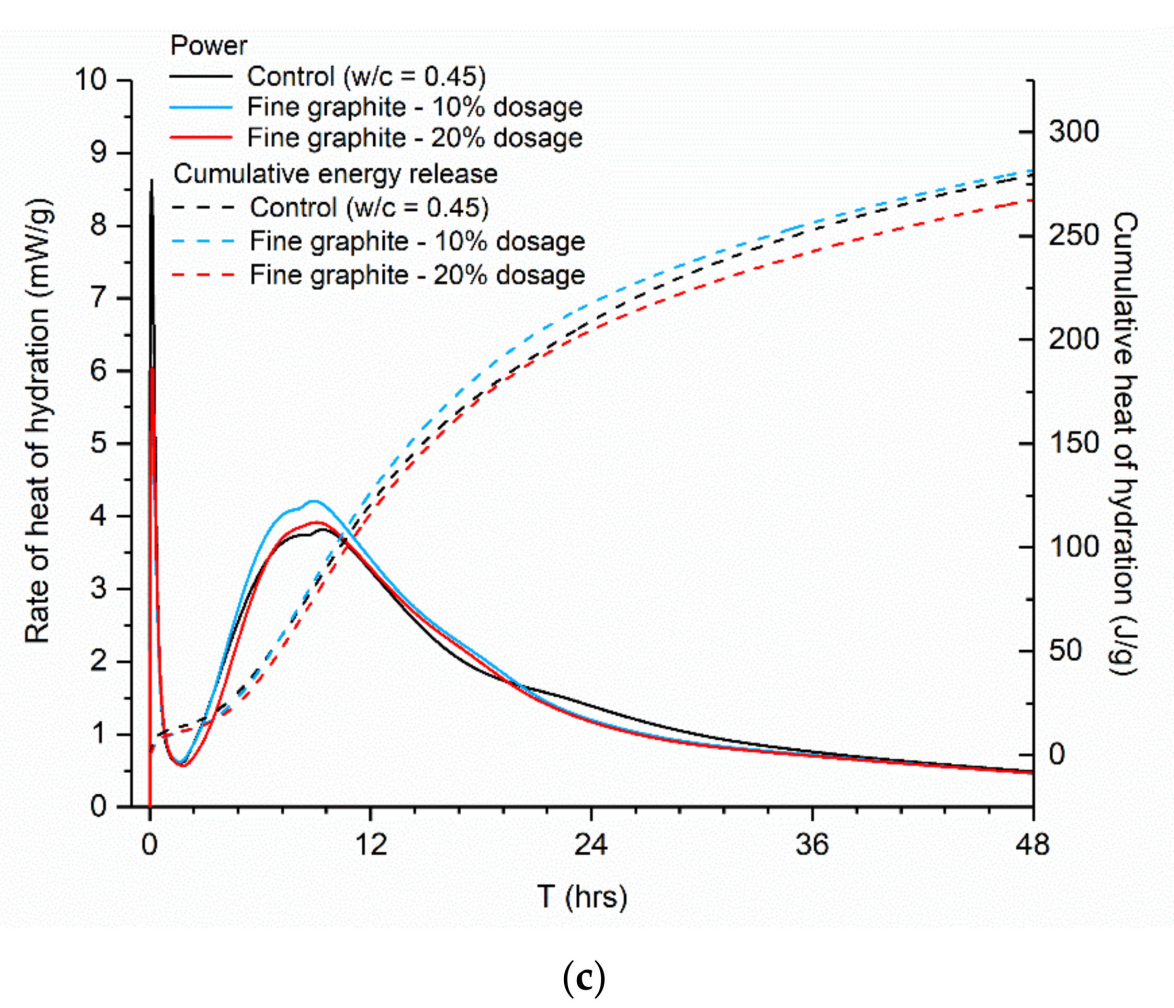
Effect of graphite size and concentration on the hydration of CEMI paste, measured by isothermal calorimetry: (**a**) coarse, (**b**) medium, and (**c**) fine graphite.

Isothermal calorimetry testing was carried out to assess the effect of graphite on the hydration of cement paste and for comparative reasons; all graphites were tested at 10 and 20 wt %, while the control (CEMI paste, *w/c* = 0.45) remained constant. The mass to what the heat/power related to in the *y*-axis (mW/g and J/g) was that of cement only. The reason that cement was chosen as the mass unit was to isolate and identify the specific contribution of the graphite content and particle size on the hydration kinetics. As illustrated in Figure 7, the same hydration peaks were observed in all cases; therefore, the cement hydration was not affected by graphite addition, in agreement to another study that showed that graphite did not directly participate in cement hydration [29]. However, the three graphite products had a somewhat different effect on the hydration. The coarse graphite depressed and widened the main hydration peak (Figure 7a), while the cumulative heat release was lower than the control. It was also observed that the hydration lines for the 10 and 20 wt % coarse graphite overlapped; hence, the increasing coarse graphite dosage did not affect the cement hydration further. These changes in the cumulative heat and progress of hydration may impact and influence the latter development of the hardened properties of the graphite-cement paste. However, the effect on the mechanical performance cannot be solely deducted from isothermal calorimetry testing; therefore, explicit mechanical testing was carried out to confirm this hypothesis and the results are presented in Section 3.4. As the graphite became finer, the effect on hydration was less pronounced. For the 10 wt % medium graphite, the impact on the hydration was insignificant (Figure 7b), and when the dosage increased to 20 wt %, the peak power increased slightly from 3.82 mW/g for the control to 4.35 mW/g. For the fine graphite (Figure 7c), the effect on hydration was even less pronounced, with the hydration curves and the total cumulative heat of hydration remaining almost unaltered, as compared to the control.

Hydration testing confirmed the hypothesis that graphite is acting as an inert filler and does not participate directly in cement hydration. The differences found between the different fineness products could be explained by a physical mechanism. The w/c was fixed, and therefore the addition of graphite resulted in an increase of the water/solids ratio. The effect of water on the hydration of Portland cement has been widely reported with higher water contents resulting in accelerated cement hydration and increased cumulative heat [11,30]. In the case of coarse graphite, the large graphite particles could have acted as physical blockers for the water to reach the cement grains and therefore resulted in a depression of the main hydration peak at ~10 h. With increasing graphite fineness, a filler effect started being present [18,31] and graphite particles helped in improving the packing density without physically blocking the water from reaching the individual cement grains. Graphite particles are also slightly hydrophobic and would push the water towards the cement grains, therefore promoting hydration. Overall, the graphite materials were not found to participate in the hydration process directly; however, the coarse graphite could block the water from reaching the cement grains, which may result in a strength reduction.

### 3.4. Mechanical Performance of Graphite-Cement Pastes

**Figure 8:**
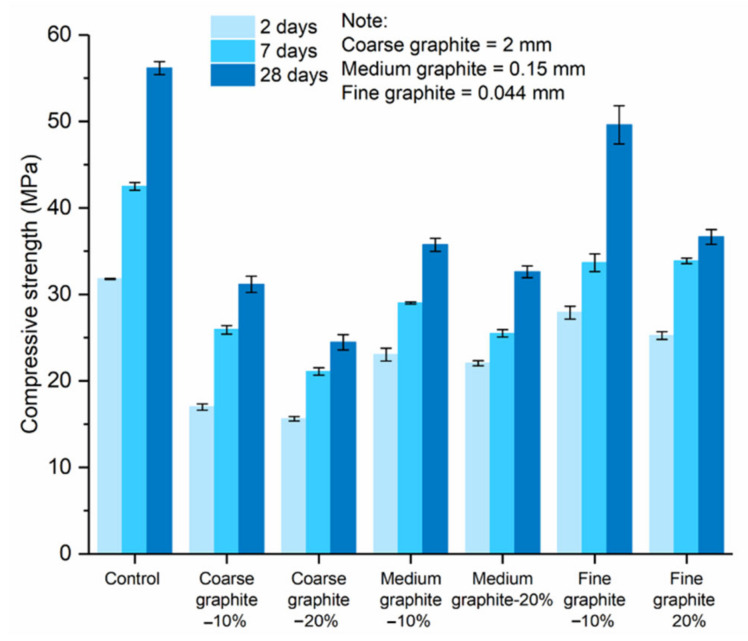
Effect of graphite fineness on the compressive strength of cement paste (*w/c* = 0.45) at 2, 7, and 28 days.

Figure 8 shows the effect of graphite fineness on the compressive strength of cement paste. The three graphite materials were used at 10 and 20 wt % concentrations. At all test ages, the compressive strength reduced with graphite, irrespective of its fineness. Furthermore, the higher the graphite dosage, the lower the compressive strength, with the 20 wt % concentration samples always resulting in a lower compressive strength than the 10 wt % samples. The summary of the % reduction in strength comparing to the control is presented in Table 2. The compressive strength had an inverse relationship with graphite size; the coarse graphite produced the lowest strength, whilst the fine graphite had the least reduction, as compared to the control.

**Figure 9:**
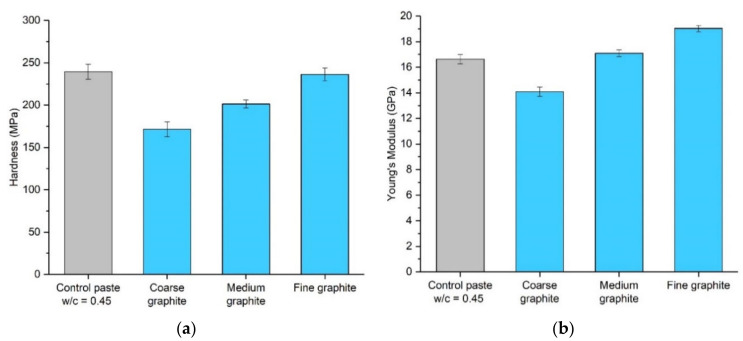
Micro-indentation results of the effect of 20 wt % graphite addition on the cement paste, at 56 days, in terms of (**a**) hardness (**b**) and Young’s modulus.

Micro-indentation testing was also carried out to assess the effect of graphite fineness on the mechanical performance of cement paste, by assessing the hardness of the specimens. Graphite–cement paste samples were tested at 56 days, with a 20 wt % graphite concentration, and the error bars show the average of 15 measurements. The compressive strength results would suggest that the hardness would reduce with graphite addition and the effects would be more pronounced for the coarse graphite. Indeed, as seen in Figure 9a, the coarser the graphite, the lower the hardness. The fine graphite almost maintained the hardness of the control specimen, while, on the contrary, the coarse graphite reduced the hardness by 28%. The results can be explained by the graphite softness, which was expected to reduce the hardness of the paste, and also because of packing density, where the finer graphite resulted in a more compact mix, which improved the overall hardness of the sample. The hardness results also provide a further indication of sufficient dispersion of graphite. The error bars were small, and the variance was similar to the control, further supporting the SEM and µCT scan findings. Young’s modulus in Figure 9b reduced for the coarse graphite by 15% but increased for the two finer graphite materials, as compared to the control (14% improvement for fine graphite). The modulus of elasticity is expected to increase with increasing compressive strength [11]; therefore, the finer the graphite, the higher the Young’s modulus (as strength was also higher). The increase in stiffness with the fine graphite could also be explained by changes in porosity, where due to better packing density, the fine graphite-cement paste had a lower porosity, and therefore it was stiffer compared to the control.

Mechanical performance testing indicated that increasing graphite fineness was beneficial for mechanical performance. The fine graphite was more effective in maintaining the compressive strength and hardness of the specimens, while the use of the coarse graphite led to significant reductions in compressive strength and hardness. Therefore, when using coarse graphite materials, the mechanical performance could be significantly compromised, and this is a key limitation in their use as conductive fillers for self-sensing application. From a practical viewpoint, it would not be feasible to use higher than 10 wt % graphite additions as the compressive strength is significantly compromised. This is particularly the case for the coarse graphite powders, that more than halved the compressive strength when added as a 20 wt % addition. To mitigate the impacts on mechanical performance, a more realistic perspective would be the targeted use of the material in locations more prone to damage. Potentially, the graphite-cement paste could be locally used as a coating of the structure rather than in the bulk of the matrix, ensuring, in this way, that the structural performance is retained, while the graphite-cement paste coating could yield the sensing capabilities, assisting with maintenance and ensuring the resilience of the structure.

### 3.5. Electrical Conductivity of Graphite-Cement Pastes

**Figure 10:**
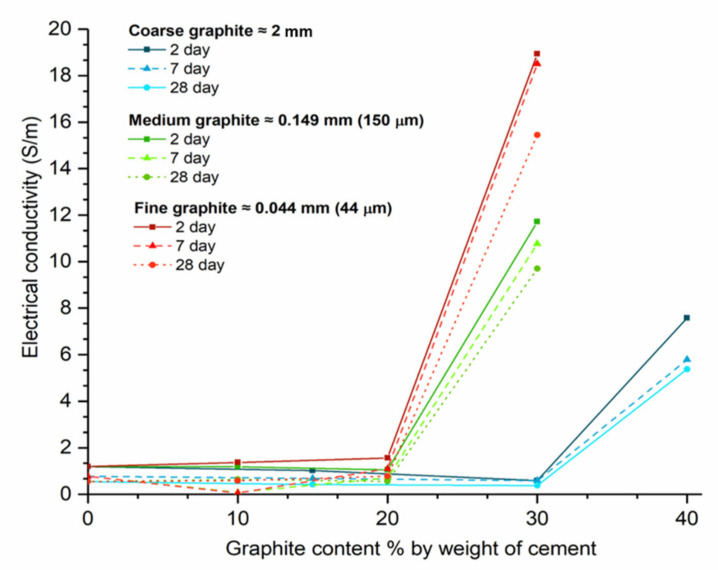
Effect of graphite fineness and dosage on the electrical conductivity of CEMI pastes (*w/c* = 0.45).

The effect of graphite fineness on the electrical conductivity of cement paste was investigated to establish whether graphite could be used for self-sensing applications. Tests were carried out at 2, 7, and 28 days, and the results in terms of electrical conductivity vs. graphite content are illustrated in Figure 10. Irrespective of graphite fineness and test age, before the sudden increase in electrical conductivity, the conductivities of all samples were less than 2 S/m. Moreover, the electrical conductivity clearly reduced as curing progressed. The two-day samples (solid lines) had higher conductivities than those tested at 7 or 28 days (dashed lines). The reason that the conductivity of the samples reduced over time was due to the free water content available in the mix. Electric current can travel both through the conductive additive (termed as electronic conduction) and through the free water available, which is termed as electrolytic conduction. As the hydration progressed from 2 to 28 days, the free water in the mix reduced; thus, it was more difficult for the electric current to pass the matrix. At the same time, at low graphite dosages, the conductive filler content was not sufficient for electronic conduction to take place and form uninterrupted travel paths. This means that, at low graphite dosages, the specimens were acting as insulators, and, over time, the electrical conductivity would diminish. Furthermore, the percolation threshold was not affected by curing age.

By observing the effect of graphite fineness, the coarse graphite (blue lines) had a percolation threshold between 30 and 40 wt % dosages, and, at 28 days, the conductivity at 40 wt % dosage was over nine times higher than the control. For the coarse graphite, it was also found that the conductivity was compromised at low graphite concentrations, and this can be explained by changes in porosity and water content in the mix. Even though the *w/c* remained constant, as graphite was added in the mix, the effective water/solids ratio was reduced, which resulted in less water available for electrolytic conduction and at the same time, the graphite concentration was not sufficient to create electric conduction paths through the specimen. Hence, the conductivity of the sample was compromised due to a reduction in the effective water in the mix. By observing the medium graphite (green lines), the percolation threshold was found at a lower concentration, compared to the coarse graphite, and at 30 wt %, the electrical conductivity was ~17 times higher, compared to the control. Therefore, the medium graphite resulted in much higher conductivity at a lower concentration, compared to the coarse graphite. For the fine graphite (red lines), the percolation threshold could be found between 20% and 30%. At 30 wt % concentration, the fine graphite had a conductivity twenty-eight times higher than the control and a 37% higher conductivity compared to the medium graphite at the same dosage.

**Figure 11:**
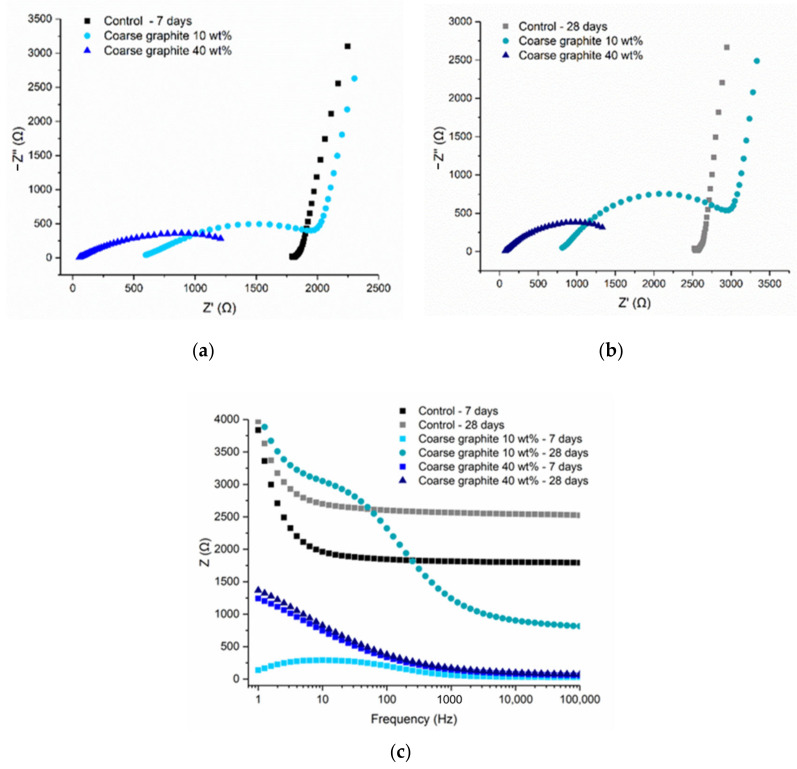
Cement paste with coarse graphite: (**a**) 7-day Nyquist plot, (**b**) 28-day Nyquist plot, and (**c**) frequency-dependent resistance.

To better understand the effect of graphite fineness on the electrical behavior of the graphite-cement pastes, electrical impedance spectroscopy (EIS) testing was undertaken. The control mix refers to a cement paste with *w/c* = 0.45, and it is the same in all cases. Coarse graphite was tested at 10 and 40 wt %, with the former being below and the latter above the percolation threshold. The Nyquist plots for 7 and 28 days are shown in Figure 11a,b, whilst the resistance vs. frequency plot is illustrated in Figure 11c. As the hydration progressed, the Nyquist plots shifted to the right, at higher true resistance values on the *x*-axis, which was expected as electrical resistance increases with curing age. The incomplete electrode arc on the right side of the plot, was very clear for the 10 wt % graphite, meaning that the resistance measurement comprised of both the inherent electrical conductivity of the material and that of the electrode. However, at 40 wt % coarse graphite (above the percolation threshold), the Nyquist plot was obviously different, and the electrode arc was not present. The measured electrical response corresponded only to the bulk response of the material, meaning that a fully conductive path was formed through the cement composites. The arc of the 40 wt % graphite remained almost unchanged as curing progressed from 7 to 28 days and no increase in resistance over time was observed. Therefore, when graphite was added at a concentration below the percolation threshold, the electrical conductivity depended on the water content, and therefore the resistivity increased over time as the hydration progressed. When the percolation threshold was exceeded, the electrolytic conduction mechanism became irrelevant and electric current traveled primarily due to the conductive network that was formed with the graphite particles. In this case, the continuous cement hydration, which reduces the free water, had an insignificant effect on the electrical resistivity.

**Figure 12:**
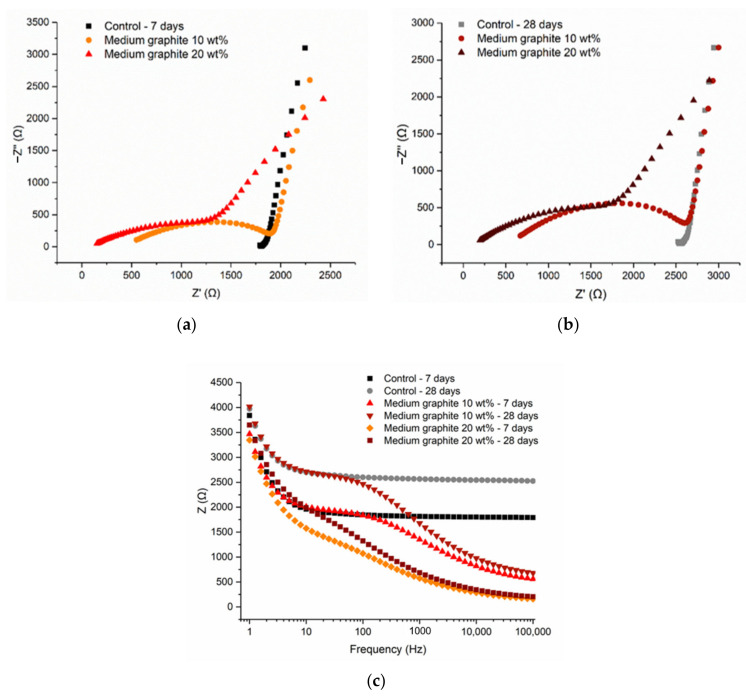
Cement paste with medium graphite: (**a**) 7-day Nyquist plot, (**b**) 28-day Nyquist plot, and (**c**) frequency-dependent resistance.

The cement pastes with the medium graphite (0.150 mm), at 10 and 20 wt %, were examined. As shown in Figure 12, the Nyquist plots at 7 and 28 days showed similar arcs, with the electrode effect present in all cases and illustrated by the incomplete rightmost arc. The resistance increased with hydration age, due to the consumption of free water, which meant that less water was available for electrolytic conduction. The fact that the arcs showed both the electrode effect and the bulk material response meant that the conductive network was not fully formed at 20 wt % and that the percolation threshold was slightly higher for this medium graphite. From Figure 12c, it can be observed that the total resistance reduced with increasing graphite content, irrespective of the frequency.

**Figure 13:**
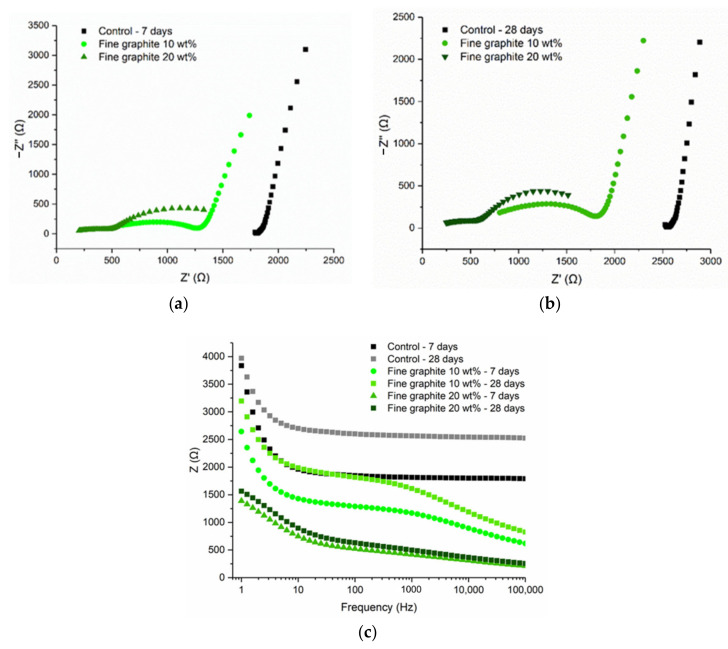
Cement paste with fine graphite: (**a**) 7-day Nyquist plot, (**b**) 28-day Nyquist plot, and (**c**) frequency-dependent resistance.

Figure 13 illustrates the findings for the fine graphite (44 µm) at 10 and 20 wt %, where the Nyquist plots between the two graphite dosages were different. The 10 wt % graphite was characterized by two arcs, a full semicircle representing the bulk material response, and an incomplete arc on the right size, which showed the electrode response to the electric current. For the 10 wt % fine graphite, the resistance increased with age due to cement hydration; therefore, the electrical conduction was due to both the presence of water (electrolytic) and conductive filler (electronic). Instead, at 20 wt %, only a semicircle arc was found at the Nyquist plots, which remained almost unchanged with age, meaning that the percolation threshold was reached. Comparing to the medium graphite, the percolation threshold was reached at 20 wt % concentration, which was not the case for the medium graphite where a higher graphite dosage was needed. Similar to the coarse and medium graphite materials, the electrical response was frequency dependent, especially below the percolation threshold. The response stabilized at ~1000 Hz for 20 wt % fine graphite but only after ~10,000 Hz for the lower graphite concentration.

The effect of graphite fineness on electrical conductivity was investigated, with an outlook that the graphite-cement pastes could be used for self-sensing applications. It was found that the coarse graphite had a percolation threshold at ~30 to 40 wt %, which reduced to between 20 and 30 wt % when a medium and a fine graphite were used. Therefore, increasing graphite fineness leads to a lower percolation threshold, which is beneficial in terms of material usage and in maintaining the mechanical performance. It should be noted that these filler dosages are higher, compared to the dosages traditionally used for inert fillers in cementitious composites. The reason that high filler dosages were selected was to ensure that an uninterrupted electrically conductive path was successfully formed in the cementitious matrix. Contrary to other inert fillers that are primarily used to improve the packing density of the mix, the purpose here was to reach the percolation threshold, which necessitated the higher additive concentrations. EIS testing showed that a combination of an electrolytic and electronic conduction mechanism was present when the conductive additive was used below the percolation threshold. Instead, when the percolation threshold was reached and an uninterrupted conductive path was formed, the electronic conduction became the dominant mechanism, and the conductivity did not depend on the presence of water. The main finding from the electrical conductivity testing was that, the finer the graphite, the lower the dosage that is needed to establish a percolation threshold, and the higher the conductivity at that dosage, as compared to a coarser graphite. These results can be explained by packing density principles. The formation of conduction paths relates to the particle size and aspect ratio of the conducting graphite filler [32]. The finer graphite particles tend to stabilize in dense configurations, resulting in more inter-particle contacts, as compared to coarser materials, creating inevitably more paths for current to pass through [33].

## 4. Conclusions

This study examined the effect of graphite fineness on the performance of cement paste to be used in self-sensing applications. Three graphite products of varying fineness were tested, and it was found that graphite fineness can greatly affect the performance of cementitious composites. In terms of early age performance, increasing graphite fineness led to a dramatic reduction in the fluidity of the paste, which could introduce practical limitations. On the other hand, graphite was not found to affect the hydration of the cement paste, meaning that it acts as an inert conductive filler. Compressive strength testing and micro-indentation showed that, the finer the graphite, the lesser the effect on mechanical performance. Electrical conductivity testing then showed that increasing graphite fineness reduces the percolation threshold for electrical conductivity. The finer graphite had a percolation threshold at 20 wt % concentration, which increased to 30–40 wt % for the coarse graphite. However, the use of graphite as a conductive additive in cement paste introduces some practical limitations. The reduction in fluidity could be a significant barrier for using this composite material in situ, while the reductions in compressive strength could make its use prohibiting for structural applications. Overall, it is recommended to use a graphite of greater fineness, as this leads to better maintenance of mechanical performance, and it reduces the dosage required to reach an electrical percolation threshold. Practical limitations around the fluidity of the paste could be overcome by adjusting the mix design and dispersion protocol.

## Figures and Tables

**Table 1:** Chemical composition of the CEMI.

Oxides (%)	CEMI 52.5N
CaO	63.4
SiO_2_	20.4
Al_2_O_3_	4.7
Fe_2_O_3_	2.7
MgO	1.0
K_2_O	0.6
SO_3_	3.1
Cl	0.02

**Table 2:** Reduction in compressive strength (%) with the three different graphite materials.

Graphite and Dosage %	2 Days	7 Days	28 Days
Coarse graphite–10%	−47%	−39%	−45%
Coarse graphite–20%	−51%	−50%	−56%
Medium graphite–10%	−28%	−32%	−36%
Medium graphite–20%	−31%	−40%	−42%
Fine graphite–10%	−12%	−21%	−12%
Fine graphite–20%	−21%	−20%	−35%
